# Medical gaslighting: navigating patient-clinician mistrust in healthcare

**DOI:** 10.3389/frhs.2025.1633672

**Published:** 2025-11-20

**Authors:** Marco Faytong-Haro

**Affiliations:** 1 Facultad de Ciencias de la Salud and School of International Studies, Universidad Espíritu Santo, Samborondón, Guayas, Ecuador; 2 Department of Sociology and Criminology & Population Research Institute, Pennsylvania State University, University Park, PA, United States

**Keywords:** medical gaslighting, patient trust, healthcare bias, safety culture, structured decision tools

## Introduction

Medical gaslighting refers to situations in which healthcare professionals dismiss, minimize, or doubt a patient's symptoms and concerns without appropriate evaluation (1). This colloquial term, derived from the concept of “gaslighting” in psychology, has gained prominence as patients share stories of feeling ignored or belittled by their providers. Such experiences can severely undermine the trust that is fundamental to the patient-clinician relationship. In recent years, the medical community has begun to acknowledge medical gaslighting as a serious problem (2).

In practice, medical gaslighting can take many forms. A clinician might interrupt a patient's description of symptoms, attribute physical complaints to stress or anxiety without evidence, or insist “it's all in your head” when diagnostic tests are inconclusive. Patients at the receiving end of these behaviors often feel disbelieved and may begin to question their own perceptions of health. Over time, gaslighting erodes trust in the patient-clinician relationship and can lead to patient safety issues when real medical conditions are brushed aside.

## Impact on patients and trust

When patients feel their concerns are dismissed, the consequences can be profound. Erosion of trust is one immediate effect, a patient who perceives that their doctor is not listening or not taking them seriously will struggle to trust that provider's guidance. This mistrust often extends to the healthcare system more broadly if multiple clinicians repeat the pattern. Patients frequently experience emotional distress in these situations, including feelings of frustration and self-doubt. Gaslighting can even lead patients to question their own sanity, causing significant psychological harm (3).

The consequences extend beyond trust: dismissed symptoms can result in missed or delayed diagnoses, sometimes for significant conditions. For example, patients with long COVID, a persistent post-COVID-19 syndrome, often encountered skepticism from clinicians. Early in the pandemic, many were told their debilitating fatigue, pain, or cognitive issues were “just stress” or anxiety. This dismissal contributed to delays in proper diagnosis and treatment for these patients (4). More generally, when a patient's complaints are written off without proper investigation, warning signs may be overlooked. In some cases, people become so disillusioned by repeated dismissals that they avoid seeking medical care altogether. Gaslighting could therefore drive patients away from the healthcare system, leading to untreated conditions and worsened outcomes.

## Contexts and populations prone to gaslighting

While any patient can experience medical gaslighting, certain groups and clinical scenarios are disproportionately affected. One prominent example involves racial bias. Black patients often describe having their symptoms not taken seriously by medical professionals. Black women's healthcare experiences provide an illustration of this: a recent Canadian study introduced the term “anti-Black medical gaslighting” to describe how Black women's concerns were systematically dismissed or downplayed by providers, especially during pregnancy and postpartum care (5). Participants reported that clinicians frequently ignored their complaints or pain, operating on biased assumptions that minimized Black women's voices and symptoms (5). These experiences reflect broader racial inequities in medicine. Experiencing gaslighting or discrimination could contribute to patients of color being less likely to seek care promptly and to place full trust in medical advice.

Another context in which patients' symptoms are frequently dismissed is in mental healthcare, through the phenomenon of diagnostic overshadowing. Diagnostic overshadowing occurs when physical symptoms reported by a patient with a psychiatric diagnosis or intellectual disability are misattributed to their mental health condition, leading clinicians to overlook a potential medical cause. A systematic review found that both mental health patients and providers frequently reported physical complaints being overshadowed by focus on a pre-existing mental illness (6). In other words, if a patient has a psychiatric label, clinicians might assume new symptoms are “just due to” that disorder instead of investigating them. For example, before long COVID was recognized as a distinct syndrome, many patients with unexplained post-COVID symptoms were presumed to have purely psychological issues and did not receive appropriate evaluation (4).

Gender bias is another driver of gaslighting in healthcare. Women's health concerns have historically been minimized, with women often labeled as overly emotional or hysterical when reporting pain. Unfortunately, this pattern persists in modern medicine. Contemporary research underscores the extent of the problem. In a 2025 study of patients with chronic vulvovaginal disorders, less than half of respondents felt their previous providers had been supportive, whereas roughly a quarter felt belittled and about one in five felt that their doctors did not believe their symptoms (7). Over half of these women had at some point considered stopping seeking medical care due to being dismissed so frequently (7). Notably, some women were even told by doctors to “just relax” or to have a glass of wine instead of receiving proper medical evaluation, a clear trivialization of women's pain (7). Conditions like endometriosis further illustrate the toll of dismissive attitudes. Despite endometriosis affecting roughly 10% of women, patients wait on average about seven years after initial symptom onset to get a diagnosis (8). This prolonged diagnostic delay is due in part to physicians normalizing women's menstrual pain or misattributing severe symptoms to benign causes, rather than investigating them rigorously (8).

A broad quantitative literature underscores how bias shapes care and outcomes. In cardiac care, women treated by female physicians had higher survival than those treated by male physicians (9), and experimental vignette studies show that physicians' catheterization recommendations varied by patient race and sex (10). Large-scale analyses confirm these patterns: women hospitalized with acute myocardial infarction (heart attack) were less likely than men to receive catheterization and often had poorer outcomes (11). Additional experimental and observational work also reveals racial bias in pain assessment and treatment linked to false biological beliefs (12). Socioeconomic bias matters too: contrary to a common perception, poorer patients are less likely to sue physicians (13). Together, these findings situate medical gaslighting within a larger evidence base on measurable bias and its consequences.

## Contributing factors to gaslighting in healthcare

Medical gaslighting rarely stems from outright malice; more often it is a byproduct of systemic issues and cognitive biases in healthcare. One major contributing factor is implicit bias. As the examples of Black women and dismissed women's pain illustrate, unconscious stereotypes about race or gender can lead providers to tune out or trivialize patients' complaints (5, 7).

Time pressure and workload are another important factor. Modern healthcare is fast-paced, and clinicians under time pressure might jump to quick conclusions, for example, assuming “nothing serious” is behind a patient's symptoms, rather than taking time to investigate. In a rushed visit, a provider may inadvertently interrupt or downplay a patient's concerns just to stay on schedule, thus engaging in gaslighting behavior.

A related issue is insufficient knowledge or training. When clinicians lack knowledge about a condition, they might dismiss symptoms rather than admit uncertainty or seek help. In the vulvovaginal disorders study, “lack of clinician knowledge” was a common theme in patients' negative encounters (7). Better medical education and humility could prevent such cases: for example, a doctor unfamiliar with a rare pain syndrome should acknowledge their limits and refer the patient to a specialist. Without such humility, the default may be to tell the patient “it's nothing” when something has simply been missed.

Another systemic contributor is culture and authority gradients. Beyond individual bias, hierarchical “authority gradients” and blame-oriented climates can silence uncertainty, inhibit speaking up, and delay referrals. These conditions that can enable gaslighting. Evidence shows that fear of appearing incompetent and negative responses from leaders are key barriers, while inclusive leadership and team training improve communication (14). In patient-safety science, this problem is often described through the contrast between Safety-I (a reactive approach focused on preventing things from going wrong) and Safety-II (a proactive approach emphasizing learning and ensuring things go right). Adopting a Safety-II mindset in healthcare helps normalize uncertainty and inquiry, especially for trainees (14–16).

Finally, clinician burnout and stress contribute to the gaslighting problem. A physician who is exhausted or emotionally depleted may have diminished capacity for empathy. Frustration or cynicism born of burnout can lead providers to become impatient with patients who have complex, hard-to-diagnose problems. Although burnout does not excuse dismissive care, healthcare leaders must address it to foster the patience and attentiveness good care requires.

## Addressing medical gaslighting and rebuilding trust

Tackling medical gaslighting requires effort on multiple fronts, aimed at changing clinician behavior, empowering patients, and improving systemic conditions. First, healthcare professionals must commit to better communication and listening. Clinicians should practice active listening, allowing patients to fully express their concerns, and show they take those concerns seriously. Even when the cause of symptoms is not immediately clear, simply acknowledging a patient's pain or distress as real can validate the patient's experience and defuse the sense of being disregarded. Training in patient-centered communication and empathy should be emphasized in medical education and continuing professional development. Shared decision making can be operationalized using the three-talk model, “choice (team) talk, option talk, decision talk”, which offers a practical structure to support empowerment in routine care (17). As one commentary put it, clinicians need to consciously “turn down the flame” on medical gaslighting by checking their biases and making a concerted effort to validate patients' reported experiences (2).

Another important strategy is education and awareness to counteract biases. Current evidence shows implicit-bias training improves knowledge, skills, and attitudes; pairing training with structural supports is recommended to affect care and safety outcomes (18, 19). Hospitals and clinics can implement training on implicit bias, cultural competency, and trauma-informed care. These interventions may help clinicians recognize their potential prejudices and understand how dismissive behaviors affect patients. For example, greater awareness about conditions commonly subject to gaslighting, such as endometriosis, would equip providers to avoid reflexively trivializing symptoms. Incorporating patient perspectives into provider training, through patient speakers or testimonials, can also humanize the issue and remind clinicians that behind every symptom is a person seeking help.

From a systems perspective, structural changes in healthcare delivery can reduce opportunities for gaslighting. Building a learning (not blame) culture is essential. Patient-safety research often frames this as moving from reactive Safety-I (“as few things as possible go wrong”) to proactive Safety-II (“as many things as possible go right”), which fosters psychological safety, reporting, and continuous learning (14).

These principles have been embedded in widely used quality-improvement tools. For example, the AHRQ Hospital Survey on Patient Safety Culture (HSOPS v2.0) provides a validated way to assess organizational culture, while the TeamSTEPPS program offers structured, evidence-based team-training strategies to strengthen communication and reduce hierarchy-related barriers (15, 20, 21).

Together, these levers help normalize speaking up and invite uncertainty, especially from trainees. In practice, organizations can assess ward or team safety culture at baseline and repeat intervals using HSOPS v2.0 (20). They can also audit the speaking-up climate and escalation/referral patterns to track whether concerns are voiced and acted upon. The training environment can be improved through TeamSTEPPS practices such as briefings, huddles, and closed-loop communication, alongside respectful ward rounds and rotating facilitation to reduce hierarchy (21). Finally, senior staff must model inclusive behaviors by explicitly inviting uncertainty, acknowledging their own limits, and praising appropriate escalation.

Allowing more time for patient appointments, especially for those with complex issues, would enable providers to investigate concerns more thoroughly rather than rushing to premature conclusions. Enhancing continuity of care, so that patients see the same clinician over time, can help build mutual trust and context, making it less likely that a patient's report will be dismissed due to unfamiliarity. In some cases, policy interventions may be warranted. For instance, to address the delays in recognizing conditions like endometriosis, experts have proposed clarifying diagnostic criteria and incentivizing early screening or specialist referral (8). By implementing clearer protocols and guidelines, healthcare organizations can ensure that reported symptoms are followed up appropriately, rather than being dismissed as inconsequential.

Another strategy is to use structured decision tools, while carefully auditing them for bias. Checklists and decision rules can reduce unwarranted variation and support team communication (e.g., the WHO Surgical Safety Checklist reduced complications and deaths across diverse hospitals) (22). However, algorithmic/AI-guided tools may encode historical inequities if trained on biased data; deployment should include upfront fairness evaluation and prospective monitoring to prevent harm (23, 24).

Empowering patients is another vital aspect of the solution. Patients who feel their concerns are not heard should be encouraged to seek second opinions or to bring an advocate (such as a family member or patient advocate) to appointments. While the onus should not be on patients to prove that they are ill, public awareness about medical gaslighting can help patients feel validated and more confident in asserting their needs.

Finally, addressing clinician well-being and the clinical environment is essential to reducing gaslighting. Better staffing and healthier work conditions are consistently associated with safer care and improved outcomes (e.g., lower mortality and failure-to-rescue) and fewer safety incidents (25, 26). Physician burnout has been linked to poorer quality interactions with patients. A recent survey found that doctors who experienced mistreatment or discrimination from patients were significantly more likely to exhibit signs of burnout (27). Burnout, in turn, erodes clinicians' empathy and patience. Healthcare organizations must therefore strive to create a culture of mutual respect, both by educating patients (and their families) to treat healthcare staff with courtesy, and by supporting providers through measures like counseling services, balanced workloads, and strong policies against abuse. Ultimately, a healthier work environment for providers translates to more empathetic, attentive care for patients, reducing the risk of gaslighting and helping to rebuild trust.
